# Author Correction: Intra-islet α-cell Gs signaling promotes glucagon release

**DOI:** 10.1038/s41467-024-50810-2

**Published:** 2024-07-29

**Authors:** Liu Liu, Kimberley EI, Diptadip Dattaroy, Luiz F. Barella, Yinghong Cui, Sarah M. Gray, Carla Guedikian, Min Chen, Lee S. Weinstein, Emily Knuth, Erli Jin, Matthew J. Merrins, Jeffrey Roman, Klaus H. Kaestner, Nicolai Doliba, Jonathan E. Campbell, Jürgen Wess

**Affiliations:** 1 Molecular Signaling Section, LBC, National Institute of Diabetes and Digestive and Kidney Diseases, Bethesda, MD 20892 USA; 2 Duke Molecular Physiology Institute, Duke University, Durham, NC 27701 USA; 3 Metabolic Diseases Branch, National Institute of Diabetes and Digestive and Kidney Diseases, Bethesda, MD 20892 USA; 4 Division of Endocrinology, Diabetes and Metabolism, Department of Medicine, University of Wisconsin-Madison, Madison, WI 53705 USA; 5 Institute for Diabetes, Obesity and Metabolism, Perelman School of Medicine, University of Pennsylvania, Philadelphia, PA 19104 USA

Correction to: *Nature Communications* 10.1038/s41467-024-49537-x, published online 15 June 2024

The original version of this Article omitted from the author list the 6th author Sarah M. Gray, who is from the ‘Duke Molecular Physiology Institute’. Consequently, the following was added to the Acknowledgements: ‘S.M.G. was supported by an NIH training grant (F32DK121420).’ Additionally, a sentence in the Author contributions was changed from ‘L.L., K.E., D.D., L.B., Y.C., C.G., E.K., E.J., M.J.M., J.R., N.D. and J.E.C. carried out experiments and interpreted and analyzed experimental data.’ to ‘L.L., K.E., D.D., L.B., Y.C., S.G., C.G., E.K., E.J., M.J.M., J.R., N.D. and J.E.C. carried out experiments and interpreted and analyzed experimental data.’ In addition, this Article contained an error in the spelling of the author Kimberley El, which was incorrectly given as E. I. Kimberley. These errors have been corrected in both the PDF and HTML versions of the Article.

