# Supplementary material for: Intra-islet α-cell Gs signaling promotes glucagon release
Source: Nat Commun. 2024 Jun 15;15:5129. doi: 10.1038/s41467-024-49537-x (PMC11180188; doi:10.1038/s41467-024-49537-x)
Supplement: Supplementary file 1 — Supplementary Information [file 41467_2024_49537_MOESM1_ESM.pdf]

Supplemental Information

Intra-islet α-cell Gs signaling promotes glucagon release

Liu Liu[1], Kimberley El[2], Diptadip Dattaroy[1], Luiz F. Barella[1], Yinghong Cui[1], Carla Guedikian[2], Min Chen[3], Lee S. Weinstein[3], Emily Knuth[4], Erli Jin[4], Matthew J. Merrins[4], Jeffrey Roman[5], Klaus H. Kaestner[5], Nicolai Doliba[5], Jonathan E. Campbell[2], Jürgen Wess[1]

[1]Molecular Signaling Section, LBC, National Institute of Diabetes and Digestive and Kidney Diseases, Bethesda, MD 20892, USA

[2]Duke Molecular Physiology Institute, Duke University, Durham, NC 27701, USA

[3]Metabolic Diseases Branch, National Institute of Diabetes and Digestive and Kidney Diseases, Bethesda, MD 20892, USA

[4]Division of Endocrinology, Diabetes and Metabolism, Department of Medicine, University of Wisconsin-Madison, Madison, WI 53705, USA

[5]Institute for Diabetes, Obesity and Metabolism, Perelman School of Medicine, University of Pennsylvania, Philadelphia, PA 19104, USA

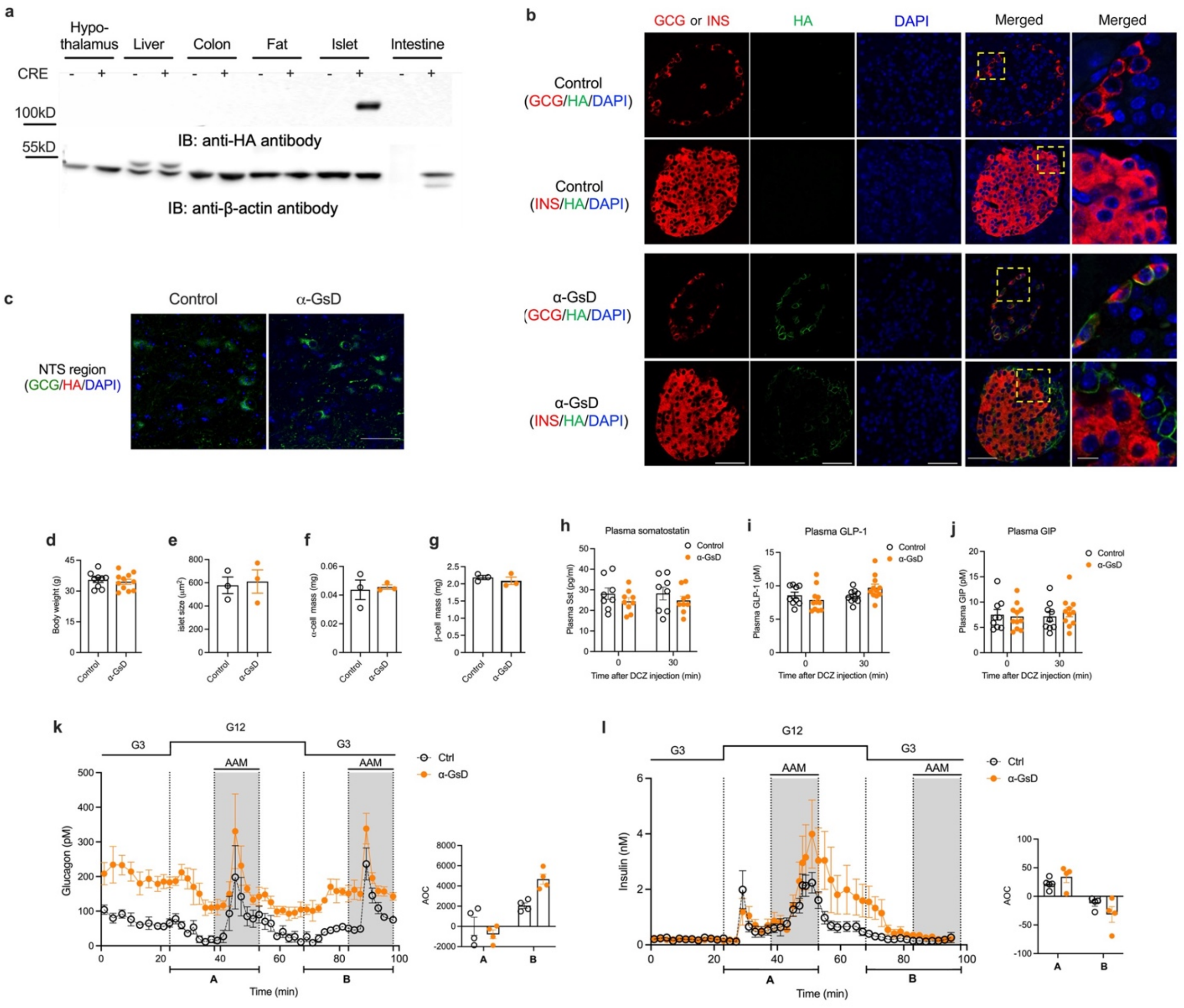

Supplementary Fig. 1. **α-GsD mice: model confirmation and basal metabolic measurements.** Selective expression of the GsD designer receptor in mouse pancreatic α-cells. (**a**) Western blot analysis detecting the HA-epitope tag fused to GsD in lysates from the indicated tissues of α-GsD mice and control littermates. (**b**) Immunofluorescence staining of pancreatic slices from α-GsD mice and control mice (see Methods for details). Cells expressing HA-tagged GsD receptors (green stain) were not detectable in control pancreatic islets (upper two rows). In contrast, studies with islets from α-GsD mice showed that glucagon-containing α-cells (red stain), but not insulin-producing β-cells (red stain), expressed GsD designer receptors (green stain) on their cell surface (lower two rows). Column 5 shows enlarged images of the areas highlighted in column 4. Scale bars: columns 1-3: 50 μm; column 4: 10 μm. (**c**) Immunofluorescence staining of brain sections containing the region of the nucleus tractus

solitarius (NTS) of α-GsD and control mice. To visualize the HA-tagged GsD receptor, sections were co-stained stained with an anti-HA (red stain) and anti-glucagon antibodies (green stain). However, no GsD signal was observed. (**d**) Body weight of α-GsD mice and control littermates (18-week-old males on regular chow) (control, n=9; α-GsKO, n=11). (**e-g**) Measurement of different islet parameters including islet size (e), α-cell mass (f), and β-cell mass (g) (n=3 per group). (**h-j**) Measurement of plasma somatostatin and incretin levels. Male α-GsD mice and control littermates (freely fed) received a single i.p. injection of DCZ (10 µg/kg, i.p.), followed by the measurement of plasma somatostatin (h; control, n=8; α-GsKO, n=9), GLP-1 (i), and GIP (j) levels 30 min later (i, j; control, n=9; α-GsKO, n=11). **(k, l)**. Islet perifusion studies. Pancreatic islets prepared from control and α-GsD mice were perifused with the indicated glucose concentrations and amino acid mixture (AAM) in the absence of DCZ. Glucagon (k) and insulin secretion (l) were measured throughout the perifusion period (n=3 or 4 mice per group, 75-100 islets per perifusion chamber). (3-4 perifusions with 75-100 islets per perifusion chamber). Western blots and immunofluorescence images are representative of three independent experiments. AOC values were calculated to quantitate hormone release during different time periods. Pancreata and islets were collected from 18-24-week-old littermates. Data are given as means ± SEM. Scale bar = 50 µm. AOC, area of the curve. Source data are provided as a Source Data file.

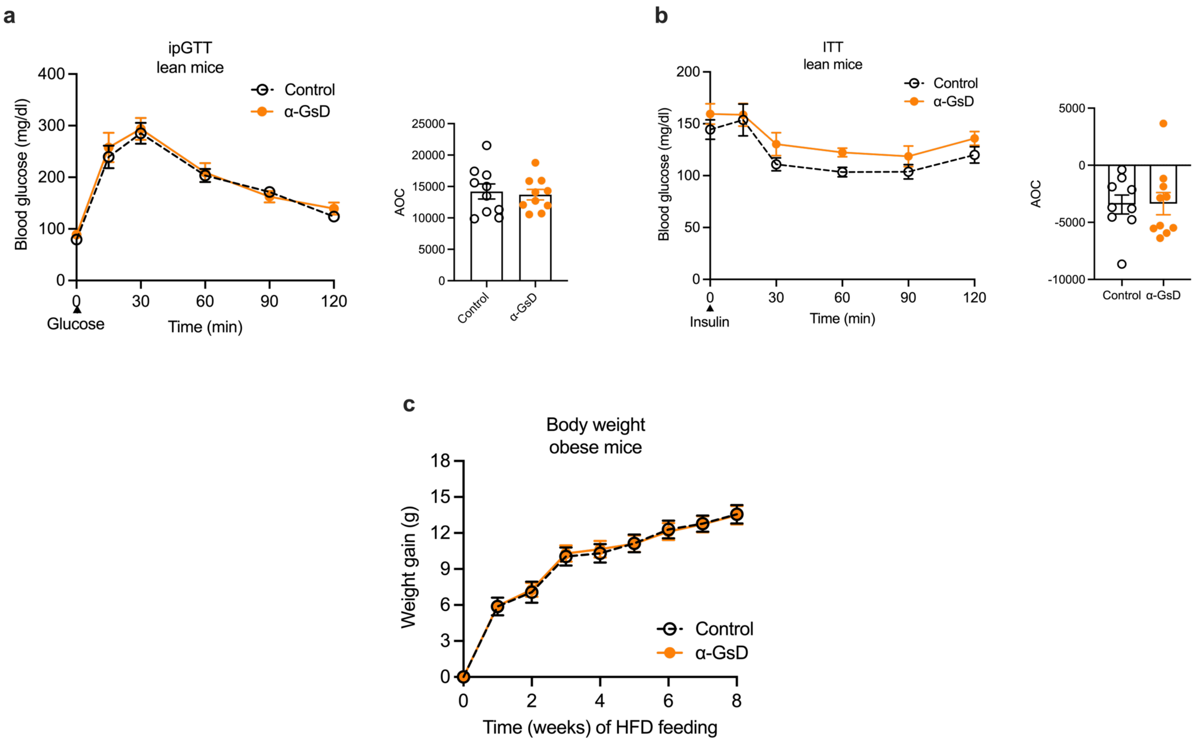

Supplementary Fig. 2. In vivo metabolic studies with α-GsD mice. (**a**) Glucose tolerance test (ipGTT). Following an overnight fast, α-GsD mice and control littermates consuming regular chow (lean mice) were injected with glucose (2 g/kg, i.p.) in the absence of DCZ. (**b**) Insulin tolerance test (ITT). Lean mice that had been fasted for 4 hr were treated with insulin (0.75 U/kg, i.p.) in the absence of DCZ. (**c**) High-fat diet (HFD) feeding causes similar increases in body weight in α-GsD and control mice during the 8 week observation period. Blood samples were taken from the tail vein. All experiments were carried out with male littermates (~20 weeks old). Data are given as means ± SEM (lean mice: control, n=10; α-GsD, n=10; mice on HFD: control, n=9; α-GsD, n=11). AOC, area of the curve. Source data are provided as a Source Data file.

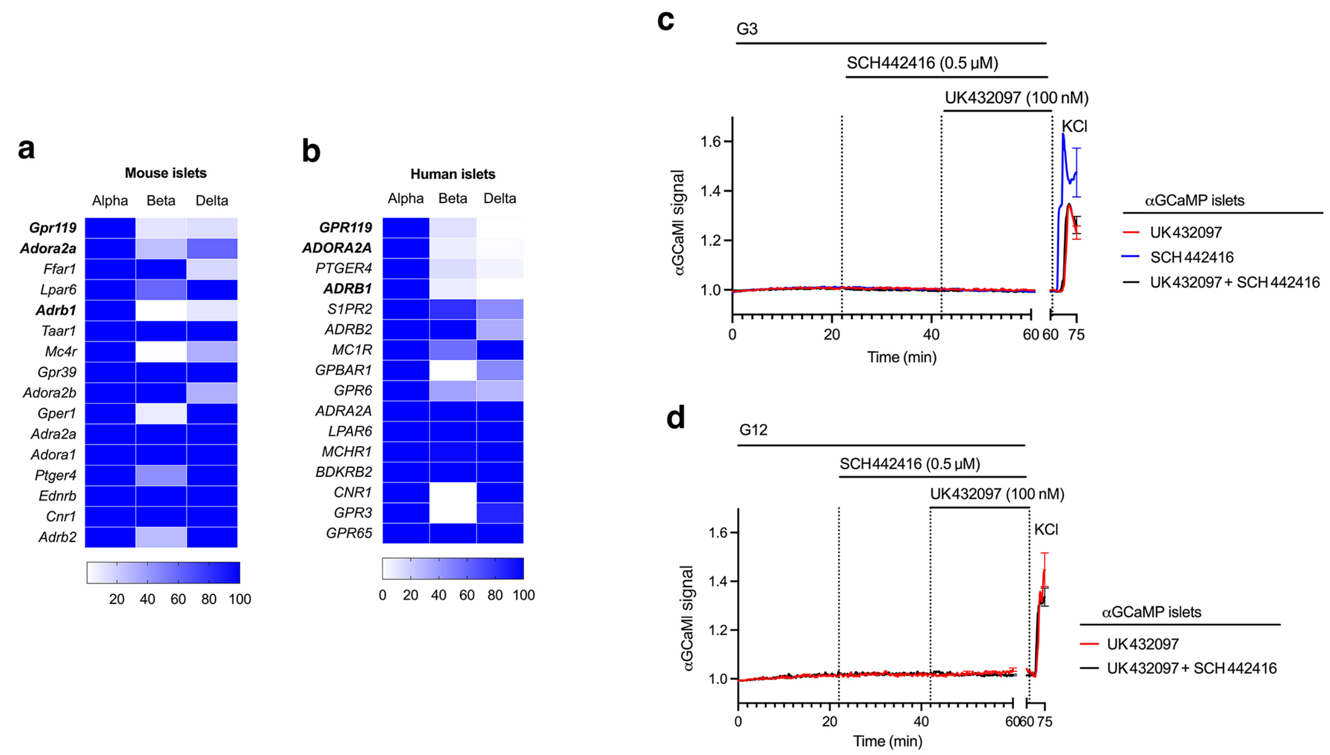

Supplementary Fig. 3. Activation of α-cell A2ARs does not affect intracellular calcium levels. (**a, b**) Expression profiles of G_s-coupled receptors in mouse and human islet cells. Analysis of public scRNAseq data [1,2] showed that the *Grp119*, *Adora2a* (encoded GPCR: A2AR) and *Adrb1* (encoded GPCR: β_1-adrenergic receptor) genes are selectively expressed at relatively high levels in both mouse (a) and human (b) α-cells. Receptors were ordered based on their expression levels in α-cells (top: highest expression). Receptor levels in other cell types were normalized relative to their expression in α-cells. (**c, d**) Ca^{2+} signals in α-cells from α-GCaMP6s mice in response to the A2AR agonist UK432097, the A2AR antagonist SCH442416, or a combination of these two ligands. Drug concentrations are given in the figure panels. KCl was used at a concentration of 30 mM. Studies were carried out with islets from α-GCaMP6s mice that express a calcium-sensitive fluorescence reporter exclusively in α-cells [3]. Data were collected at low and high glucose concentrations (G3 (b) and G12 (c), respectively) (66-80 islets per mouse; mouse age: 13 weeks). Traces represent means ± SEM (n=3 independent experiments/mice). Source data are provided as a Source Data file.

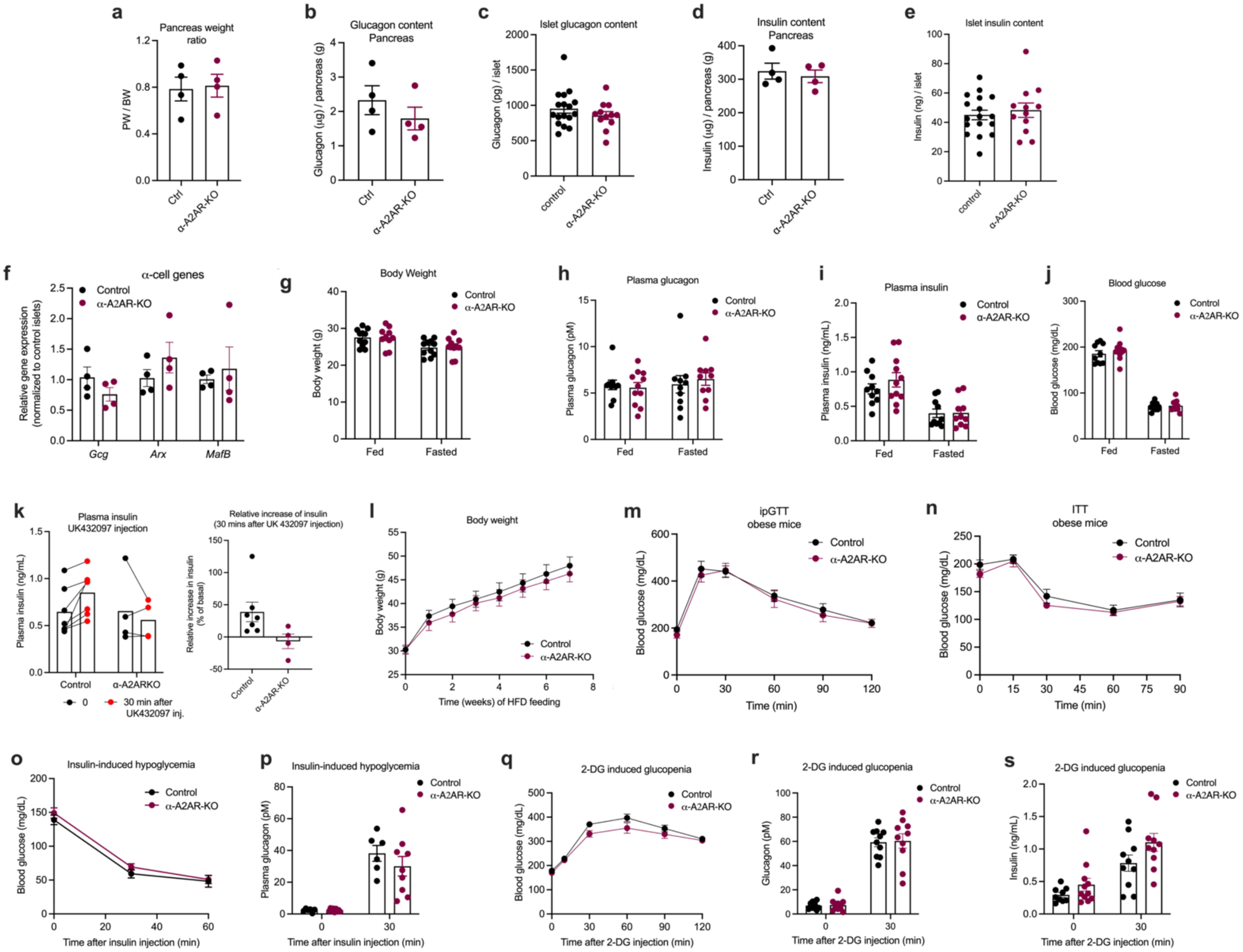

Supplementary Fig. 4. **In vitro and in vivo studies with α-A2AR-KO mice**.

(**a-f**) Measurements of pancreatic parameters of α-A2AR-KO mice and control littermates maintained on regular chow. (a) Ratio of pancreas weight (PW) to body weight (BW) (n=4 per group). (b) Pancreatic glucagon content (n=4 per group). (c) Islet glucagon content (control, n=17; α-A2AR-KO, n=12). (d) Pancreatic insulin content (n=4 per group). (e) Islet insulin content (control, n=17; α-A2AR-KO, n=12). (f) Islet α-cell gene expression analysis (*Gcg*, *Arx*, and *MafB*) (n=4 per group). (**g-j**) Body weight (g), plasma glucagon (h), plasma insulin (i), and blood glucose (j) levels of freely fed and fasted (overnight) α-A2AR-KO mice and control littermates (control, n=10 or 11; α-A2AR-KO, n=10 or 11). (k) Plasma insulin levels of α-A2AR-KO and control mice after i.p. injection of the A2AR agonist UK432097 (5 mg/kg) (control, n=7; α-A2AR-KO, n=4). (**l-n**) Metabolic tests carried out with α-A2AR-KO mice and

control littermates maintained on a high-fat diet (HFD; obese mice). (l) Body weight changes during HFD feeding. Mice were maintained on the HFD for at least 8 weeks, after having consumed regular chow for 14 weeks (control, n=9; α-A2AR-KO, n=8). (m) ipGTT. Obese mice that had been fasted overnight were injected with glucose (1 g/kg, i.p.) (n=10 per group). (n) ITT. After a 4 hr fast, obese mice were injected with insulin (1 U/kg, i.p.). Blood glucose levels were measured at the indicated post-injection time points (control, n=9; α-A2AR-KO, n=8). (**o, p**) Changes in plasma glucagon (o) and blood glucose (p) levels after i.p. injection of insulin (1 U/kg) (mouse diet: regular chow) (control, n=6; α-A2AR-KO, n=9). (**q-s**) Changes in blood glucose (q), plasma glucagon (r), and plasma insulin (s) levels after i.p. injection of 2-DG (500 mg/kg) (mouse diet: regular chow) (control, n=10; α-A2AR-KO, n=11). Blood samples were collected from the tail vein. All experiments were carried out with male littermates (12-25 weeks old). Data are given as means ± SEM. Source data are provided as a Source Data file.

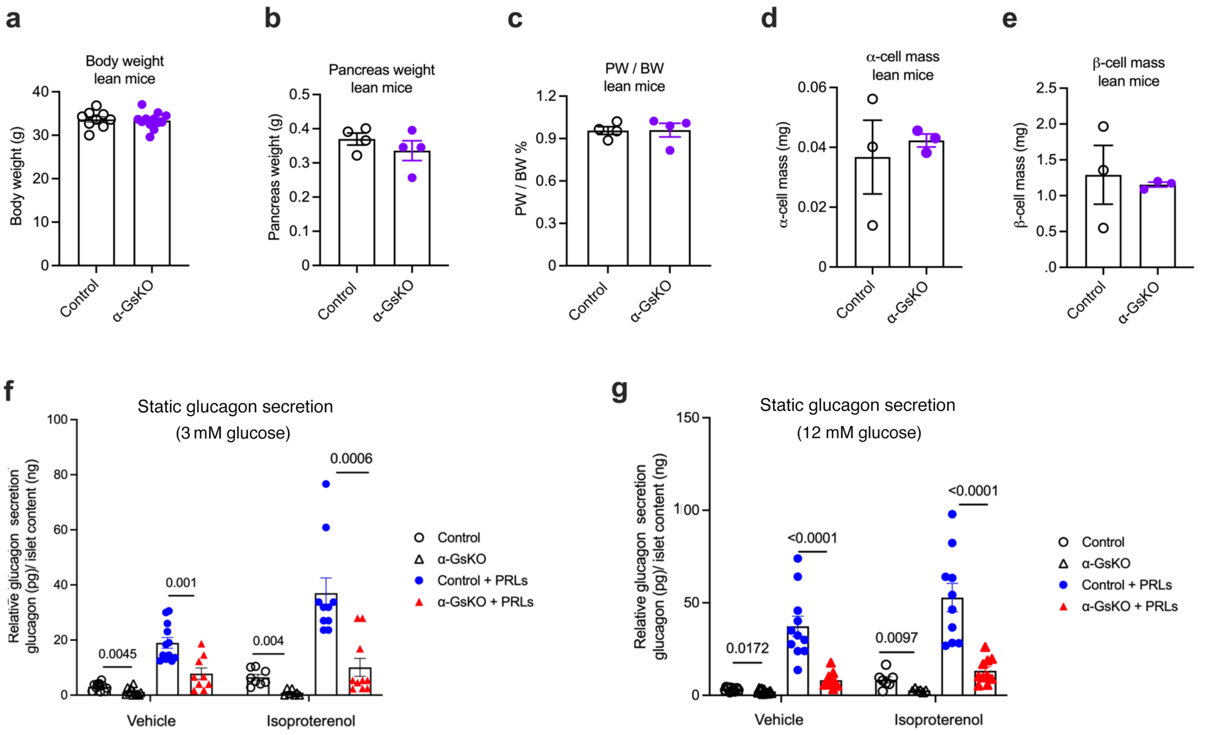

Supplementary Fig. 5. **Measurement of body weight and various pancreas-related parameters of α-GsKO mice.** In (**a-f**), measurements were performed with α-GsKO mice and control littermates consuming regular chow (lean mice). (a) Body weight (10-week-old male mice) (control, n=9 ; α-GsKO, n=12). (b) Pancreas weight. (c) Ratio of pancreas weight (PW) to body weight (BW) (b, c; n=4 per group). (d) α-cell mass. (e) β-cell mass (d, e; n=3 per group). (**f, g**) Static glucagon secretion assay in the presence of somatostatin (SST) receptor antagonists. Pancreatic islets from control and α-GsKO mice were incubated in the presence of 3 mM glucose (f) (control, n= 8-12; α-GsKO, n=7-10) or 12 mM glucose (g) (control, n= 7-12; α-GsKO, n=6-12), respectively, either in the absence or presence of SSTR antagonists (PRLs: PRL2915 + PRL3195, 1 µM each). Under these conditions, treatment of control islets with 5 µM isoproterenol (β-adrenergic receptor agonist) strongly stimulated glucagon release at both G3 (f) and G12 (g). This response was virtually abolished in α-GsKO islets. Numbers in panels (f) and (g) refer to p values. Data are given as means ± SEM. Multiple t test with Bonferroni correction was applied in panels f and g). Source data are provided as a Source Data file.

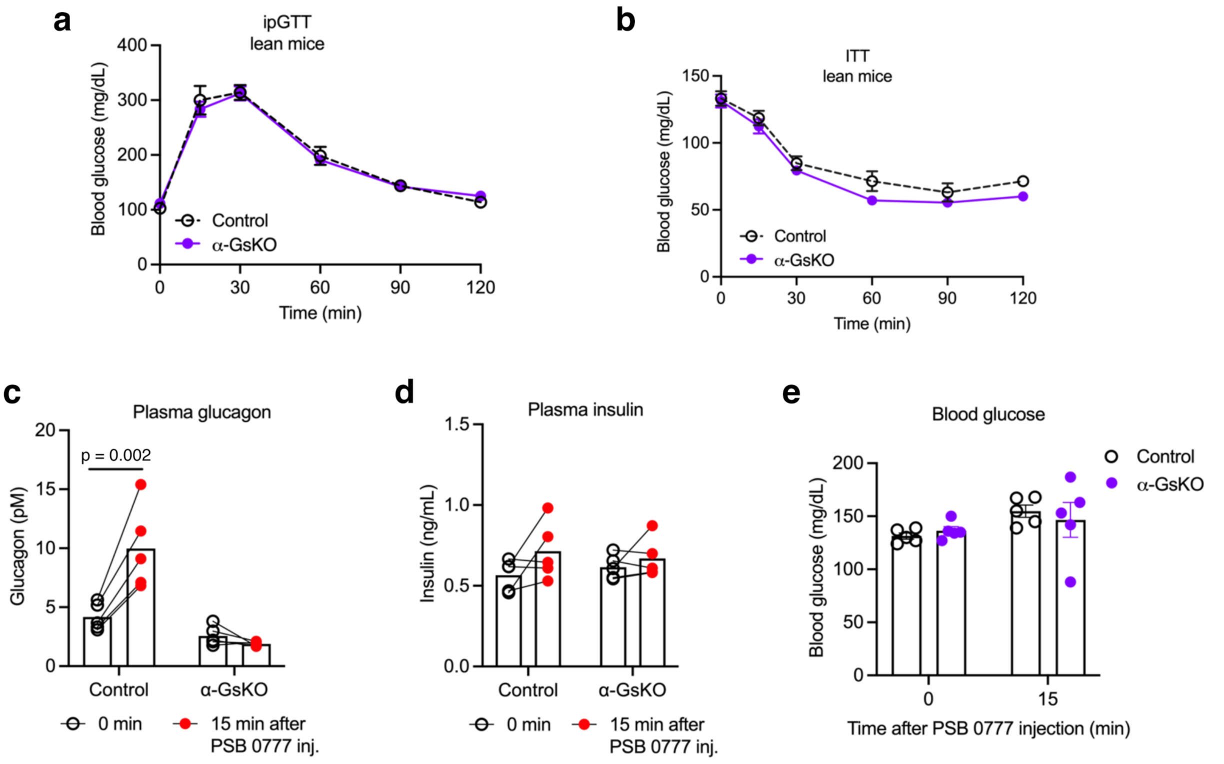

Supplementary Fig. 6. **In vivo metabolic studies with α-GsKO mic. (a, b**) In vivo metabolic studies performed with α-GsKO mice and control littermates consuming regular chow (lean mice). (a) ipGTT. Mice that had been fasted overnight were injected with glucose (2 g/kg, i.p.) (control, n= 5; α-GsKO, n= 16). (b) ITT. After a 4 hr fast, mice were injected with insulin (0.75 U/kg, i.p.) (control, n= 5; α-GsKO, n= 15). (**c-e**) Levels of plasma glucagon (c), plasma insulin (d), and blood glucose (e) after i.p. injection of PSB 0777 (A2AR agonist, 1 mg/kg) in control and α-GsKO mice (mouse diet: regular chow) (n=5 per group). Blood samples were collected from the tail vein. Data are given as means ± SEM. Data were analyzed via two-way repeated measure ANOVA for time with Bonferroni post hoc test (c). Numbers in panels (c) refer to p values. Source data are provided as a Source Data file.

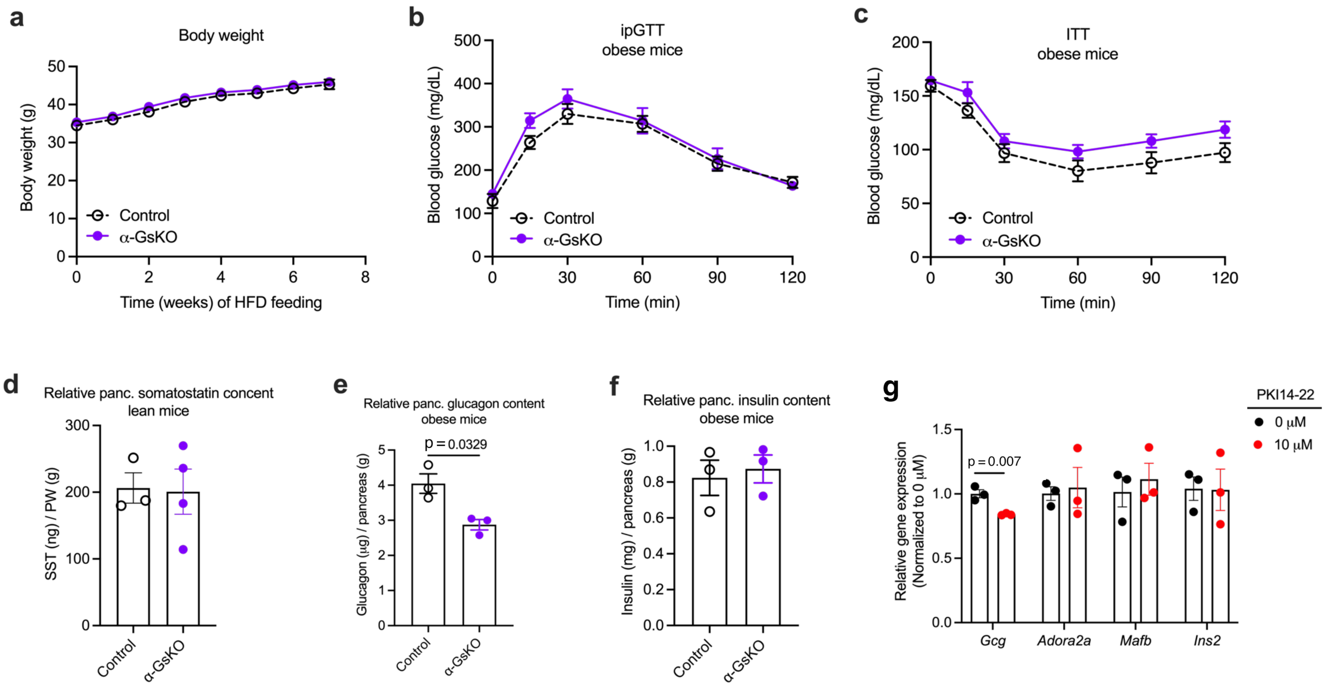

Supplementary Fig. 7. In vitro and in vivo metabolic analysis of α-GsKO mice. In vivo metabolic studies were performed with α-GsKO mice and control littermates consuming a high-fat diet (HFD, obese mice). (**a**) Body weight during HFD treatment (control, n= 12; α-GsKO, n= 10). (**b**) ipGTT. Obese mice that had been fasted overnight were injected with glucose (1 g/kg, i.p.) (control, n= 9; α-GsKO, n= 10). (**c**) ITT (obese mice). Following a 4 hr fast, mice were injected with insulin (1 U/kg, i.p.) (n= 9 per group). Blood glucose levels were measured at the indicated post-injection time points. Blood samples were taken from the tail vein. (**d-f**) Relative pancreatic somatostatin (d), glucagon (e), and insulin (f) content of α-GsKO and control mice (control, n= 3; α-GsKO, n= 3 or 4). (**g**) qRT-PCR gene expression analysis using RNA prepared from α-TC6 cells incubated in the absence or presence of a selective PKA inhibitor (PKI12-22, 10 μM) for 16 hr (n= 3 per group). All data shown in (a-f) were carried out using male littermates. Data are given as means ± SEM. Data in (d- g) were analyzed via two-tailed Student's t test. Numbers in panels (e) and (g) refer to p values. Source data are provided as a Source Data file.

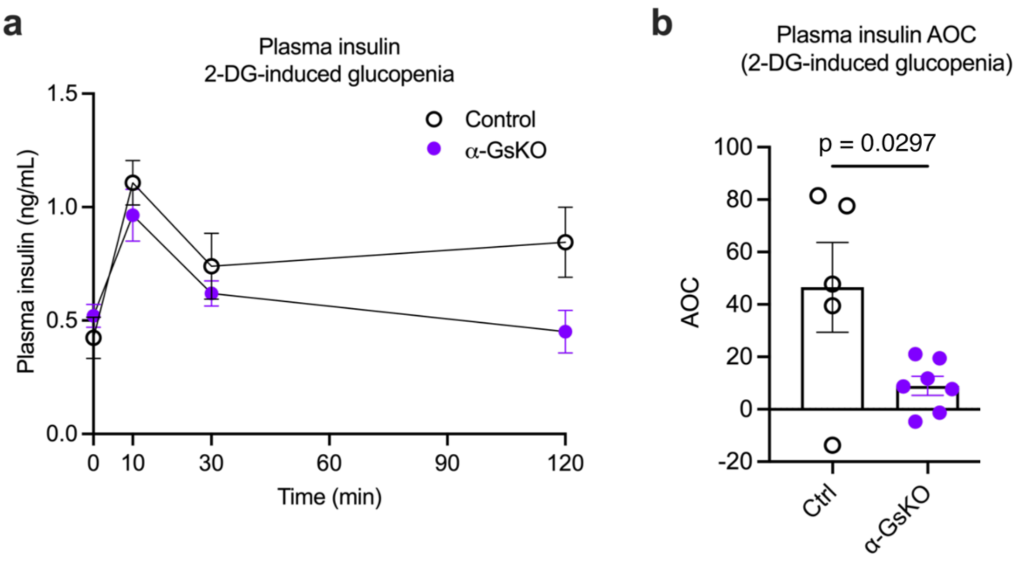

Supplementary Fig. 8. Lack of α-cell G_s signaling causes reduced insulin excursions following 2-DG treatment. (**a**) Plasma insulin levels of control and α-GsKO mice following i.p. injection of 2-DG (500 mg/kg). Mice were maintained on regular rodent chow. (**b**) AOC plot of the data shown in (a). Data are given as means ± SEM (control, n=5; α-GsKO, n=7). Data in (b) were analyzed via two-tailed Student's t test.. Numbers in panels (b) refer to p values. AOC, area of the curve. Source data are provided as a Source Data file.

Supplementary Table 1. Summary of antibodies, drugs, reagents, kits, and mouse strains used in this study

**Reagent or Resource**	**Source**	**Cat #**
**Antibody**		(Dilution used in parentheses)
Alexa Fluor 488 goat anti-guinea pig secondary Ab	Thermo Fisher Scientific	A-11073 (1:500)
Alexa Fluor 488 goat anti-mouse secondary Ab	Thermo Fisher Scientific	A-11017 (1:500)
Alexa Fluor 488 goat anti-rabbit secondary Ab	Thermo Fisher Scientific	A-11034 (1:500)
Alexa Fluor 555 goat anti-guinea pig secondary Ab	Thermo Fisher Scientific	A-21435 (1:500)
Alexa Fluor 594 goat anti-mouse secondary Ab	Thermo Fisher Scientific	A-11032 (1:500)
Alexa Fluor 594 goat anti-rabbit secondary Ab	Thermo Fisher Scientific	A-11037 (1:500)
Alexa Fluor 594 donkey anti-rabbit secondary Ab	Abcam	Ab150064 (1:1000)
Anti-mouse IgG, HRP-linked secondary Ab	Cell Signaling	7076 (1: 5000)
Anti-rabbit IgG, HRP-linked secondary Ab	Cell Signaling	7074 (1:5000)
Guinea pig anti-insulin Ab	Abcam	7842 (1:200)
Mouse anti-glucagon Ab	Abcam	ab10988 (1:200)
Mouse anti-β-actin Ab	Cell Signaling	3700 (1:1000)
Rabbit anti-$G\alpha_s$ Ab	generated in the lab of Dr. Lee S. Weinstein	(1:1000)
Rabbit anti-HA Ab	Cell Signaling	3724 (1:1000)
Rabbit anti-pCREB	Cell Signaling	9198 (1:100)
Antifade Mounting Medium with DAPI	Vector Laboratories	H-2000-10
**Compound**		
(−)- Isoproterenol hydrochloride	MilliporeSigma	I6504
2-Deoxy-D-glucose (2-DG)	MilliporeSigma	D8375
Accutase	MilliporeSigma	A6964
Adenosine deaminase	Worthington	LS009043
Aprotinin	MilliporeSigma	A3428
Bovine serum albumin (fatty acid-free)	MilliporeSigma	A7030
Collagenase from Clostridium histolyticum	MilliporeSigma	C7657
cOmplete, EDTA-free protease inhibitor cocktail	MilliporeSigma	11697498001
Corn oil	MilliporeSigma	C8267
d[Leu^4,Lys^8]-VP	Tocris	3127

Deschloroclozapine (DCZ)	Hello Bio Inc	HB8555
DPP-4 inhibitor (KR-62436)	MilliporeSigma	K4264
ECL Western blotting substrate	Thermo Fisher Scientific	32106
Histopaque 1077	MilliporeSigma	10771
Human [D-Ala^2] GIP	Tocris	6699
Human insulin (Humulin R U-100)	Eli Lilly	NDC 0002-8215-17
L-Alanine	MilliporeSigma	A7627
Normal goat serum	Vector Laboratories	S1000
PKI 14-22	Tocris	2546
PSB 0777 ammonium salt	Tocris	4334
PRL2915	Bachem	H-6056
PRL3195	Bachem	H-5884
SCH 442416	Tocris	2463
Tamoxifen	MilliporeSigma	T5648
Tween 20	Fisher Scientific	P7949
TRIzol	Invitrogen	15596026
UK 432097	Axon Medchem	1193
**Kit**		
Glucagon ELISA kit (plasma)	Mercodia	10-1281-01
Glucagon ELISA kit (plasma)	Crystal Chem	81520
Ultra-Sensitive Mouse Insulin ELISA kit (plasma)	Crystal Chem	9008
Mouse GLP-1 ELISA kit (plasma)	Crystal Chem	81508
Mouse Active GIP ELISA kit (plasma)	Crystal Chem	81511
Mouse SST ELISA kit	LS Bio	LS-F12622-1
Lumit Glucagon Immunoassay (ex vivo studies)	Promega	W8022
Lumit Insulin Immunoassay (ex vivo studies)	Promega	CS3037A01
Glucagon Quantikine ELISA Kit (ex vivo studies)	R&D System	DGCG0
BCA protein assay kit	Thermo Fisher Scientific	23225
**Experimental Models: Organisms/Strains**		
C57BL/6N mice (WT mice)	Taconic	C57BL/6NTac
Floxed *Adora2a* (*Adora2a$^{fl/fl}$*)	Mice provided by Dr. Yuqing Huo	REF[4]

Floxed *Gnas* $(G\alpha_s)$ ($Gnas^{fl/fl}$)	Mice provided by Dr. Lee Weinstein	REF[5]
CAG-LSL-GsD knock-in	Mice provided by Dr. Rebecca Berdeaux	REF[6]
$Gcg\text{-}Cre^{ERT2}$	Mice provided by Dr. Klaus H. Kaestner	REF[7]
CAMPER mice	JAX	032205
GCaMP6s mice	JAX	028866

Supplementary Table 2. Primers and TaqMan reagents used for PCR/qRT-PCR experiments

**Genes**	**Forward Sequence (5'-3')**	**Reverse Sequence (5'-3')**
Gcg-Cre^{ERT2}	CCTGGAAAATGCTTCTGTCCG	CAGGGTGTTATAAGCAATCCC
ROSA26-LSL-Gs-DREADD-CRE-luc	CTCGAAGTACTCGGCGTAGG	CTTGGCAATCCGGTACTGTT
Gnas$^{fl/fl}$	TTCGGTCTCGTCCCCTTAGTTG	AACAAATCGCACACCCCAGTGAGG
Adora2a$^{fl/fl}$	GGGCAAGATGGGAGTCATT	ATTCTGCATCTCCCGAAACC
Gcg	TTCCCAGAAGAAGTCGCCATT	GGTGCTCATCTCGTCAGAGAA
Mafb	TTCGACCTTCTCAAGTTCGACG	TCGAGATGGGTCTTCGGTTCA
Arx	GGCCGGAGTGCAAGAGTAAAT	TGCATGGCTTTTTCCTGGTCA
Ins2	CTGGCCCTGCTCTTCCTCTGG	CTGAAGGTCACCTGCTCCCGG
Mafa	AGGAGGAGGTCATCCGACTG	CTTCTCGCTCTCCAGAATGTG
Pdx1	CCCCAGTTTACAAGCTCGCT	CTCGGTTCCATTCGGGAAAGG
36b4	ATGGGTACAAGCGCGTCCTG	GCCTTGACCTTTTCAGTAAG

**Gene symbol**	**Encoded protein name**	**TaqMan Assay ID**
Adora2a	Adenosine A_{2A} receptor	Mm00802075_m1
Gcg	Glucagon	Mm01269055_m1
Gnaq	$G\alpha_q$	Mm00492381_m1
Gnas	$G\alpha_s$	Mm00507037_m1
Ins2	Insulin 2	Mm00731595_gH
Ppia	Peptidylprolyl isomerase A	Mm02342430_g1
Sst	Somatostatin	Mm00436671_m1

Supplementary Table 3. Information about human islet donors

RRID	Recovery Center	Sex	Age (years)	BMI	Race
SAMN19591106	SC-ICRC	M	61	29.3	Hispanic
SAMN19920583	Scharp-Lacy	M	54	24.5	Caucasian
SAMN20821183	Scharp-Lacy	M	58	31.7	Caucasian
SAMN21032331	Scharp-Lacy	M	40	27.4	African American
SAMN21399152	Scharp-Lacy	M	29	22.9	Asian
SAMN23009382	Scharp-Lacy	F	63	33.5	Asian
SAMN23079315	Univ. of Miami	F	37	30.2	Caucasian

References

1. Baron, M., *et al.* A Single-Cell Transcriptomic map of the human and mouse pancreas reveals inter- and intra-cell population structure. *Cell Syst* **3**, 346-360.e344 (2016).
2. DiGruccio, M.R., *et al.* Comprehensive alpha, beta and delta cell transcriptomes reveal that ghrelin selectively activates delta cells and promotes somatostatin release from pancreatic islets. *Mol Metab* **5**, 449-458 (2016).
3. El, K., *et al.* GIP mediates the incretin effect and glucose tolerance by dual actions on α cells and β cells. *Sci Adv* **7,** eabf1948 (2021).
4. Liu, Z., *et al.* Endothelial adenosine A2a receptor-mediated glycolysis is essential for pathological retinal angiogenesis. *Nature Commun* **8**, 584 (2017).
5. Chen, M., *et al.* Gsα deficiency in skeletal muscle leads to reduced muscle mass, fiber-type switching, and glucose intolerance without insulin resistance or deficiency. *Am J Physiol - Cell Physiol* **296**, C930-C940 (2009).
6. Akhmedov, D., *et al.* Gs-DREADD knock-in mice for tissue-specific, temporal stimulation of cyclic AMP signaling. *Mol Cell Biol* **37**, e00584-00516 (2017).

7. Ackermann, A.M., Zhang, J., Heller, A., Briker, A. & Kaestner, K.H. High-fidelity Glucagon-CreER mouse line generated by CRISPR-Cas9 assisted gene targeting. *Mol Metab* **6**, 236-244 (2017).
