# Supplementary material for: Intra-islet α-cell Gs signaling promotes glucagon release
Source: Nat Commun. 2024 Jun 15;15:5129. doi: 10.1038/s41467-024-49537-x (PMC11180188; doi:10.1038/s41467-024-49537-x)
Supplement: Supplementary file 3 — Reporting Summary [file 41467_2024_49537_MOESM3_ESM.pdf]

nature portfolio

Corresponding author(s): Jürgen Wess, PhD

Last updated by author(s): 05/27/2024

Reporting Summary

Nature Portfolio wishes to improve the reproducibility of the work that we publish. This form provides structure for consistency and transparency in reporting. For further information on Nature Portfolio policies, see our Editorial Policies and the Editorial Policy Checklist.

Statistics

For all statistical analyses, confirm that the following items are present in the figure legend, table legend, main text, or Methods section.

n/a	Confirmed	
☐	☒	The exact sample size (*n*) for each experimental group/condition, given as a discrete number and unit of measurement
☐	☒	A statement on whether measurements were taken from distinct samples or whether the same sample was measured repeatedly
☐	☒	The statistical test(s) used AND whether they are one- or two-sided<br>*Only common tests should be described solely by name; describe more complex techniques in the Methods section.*
☐	☒	A description of all covariates tested
☐	☒	A description of any assumptions or corrections, such as tests of normality and adjustment for multiple comparisons
☐	☒	A full description of the statistical parameters including central tendency (e.g. means) or other basic estimates (e.g. regression coefficient) AND variation (e.g. standard deviation) or associated estimates of uncertainty (e.g. confidence intervals)
☐	☒	For null hypothesis testing, the test statistic (e.g. *F*, *t*, *r*) with confidence intervals, effect sizes, degrees of freedom and *P* value noted<br>*Give P values as exact values whenever suitable.*
☒	☐	For Bayesian analysis, information on the choice of priors and Markov chain Monte Carlo settings
☒	☐	For hierarchical and complex designs, identification of the appropriate level for tests and full reporting of outcomes
☒	☐	Estimates of effect sizes (e.g. Cohen's *d*, Pearson's *r*), indicating how they were calculated

Our web collection on statistics for biologists contains articles on many of the points above.

Software and code

Policy information about availability of computer code

Data collection	Prism9, Excel
Data analysis	Prism9, Excel, ImageJ

For manuscripts utilizing custom algorithms or software that are central to the research but not yet described in published literature, software must be made available to editors and reviewers. We strongly encourage code deposition in a community repository (e.g. GitHub). See the Nature Portfolio guidelines for submitting code & software for further information.

Data

Policy information about availability of data

All manuscripts must include a data availability statement. This statement should provide the following information, where applicable:

- Accession codes, unique identifiers, or web links for publicly available datasets
- A description of any restrictions on data availability
- For clinical datasets or third party data, please ensure that the statement adheres to our policy

Source data for all figures will be provided with the paper. All other data supporting the findings of this study are available from the authors upon request.

Research involving human participants, their data, or biological material

Policy information about studies with human participants or human data. See also policy information about sex, gender (identity/presentation), and sexual orientation and race, ethnicity and racism.

Reporting on sex and gender	No human participants are involved in this study.
Reporting on race, ethnicity, or other socially relevant groupings	No human participants are involved in this study.
Population characteristics	No human participants are involved in this study.
Recruitment	No human participants are involved in this study.
Ethics oversight	No human participants are involved in this study.

Note that full information on the approval of the study protocol must also be provided in the manuscript.

Field-specific reporting

Please select the one below that is the best fit for your research. If you are not sure, read the appropriate sections before making your selection.

[x] Life sciences [ ] Behavioural & social sciences [ ] Ecological, evolutionary & environmental sciences

For a reference copy of the document with all sections, see nature.com/documents/nr-reporting-summary-flat.pdf

Life sciences study design

All studies must disclose on these points even when the disclosure is negative.

Sample size	Sample size was chosen based on prior experience of the investigators with similar experiments previously published. The authors have published numerous peer-reviewed papers demonstrating clear positive findings with similar sample sizes for the types of experiments included.
Data exclusions	No data points were excluded from the analysis of any of the experiments.
Replication	All experimental findings were reproduced in several independent experiments, as indicated in the figure legends.
Randomization	Randomization was performed by blinding investigators to genotype and allowing them to choose each subject blindly.
Blinding	Randomization was performed by blinding investigators to genotype and allowing them to choose each subject blindly. For studies using pharmacological agents, the investigator was aware of the agent being used, but was not aware of the genotypes of the animals used. Investigators were not aware of the specific group to which an animal was assigned to when doing the experiment or until after completion of the experiment.

Reporting for specific materials, systems and methods

We require information from authors about some types of materials, experimental systems and methods used in many studies. Here, indicate whether each material, system or method listed is relevant to your study. If you are not sure if a list item applies to your research, read the appropriate section before selecting a response.

Materials & experimental systems

n/a	Involved in the study	
[ ]	[x]	Antibodies
[ ]	[x]	Eukaryotic cell lines
[x]	[ ]	Palaeontology and archaeology
[ ]	[x]	Animals and other organisms
[x]	[ ]	Clinical data
[x]	[ ]	Dual use research of concern
[x]	[ ]	Plants

Methods

n/a	Involved in the study	
[x]	[ ]	ChIP-seq
[x]	[ ]	Flow cytometry
[x]	[ ]	MRI-based neuroimaging

Antibodies

Antibodies used	The following primary antibodies were used:<br>Guinea pig anti-insulin Ab(Abcam, #7842) (1:200)

Mouse anti-glucagon Ab (Abcam, # ab10988) (1:200)
Mouse anti-β-actin Ab(Cell Signaling, # 3700) (1:1000)
Rabbit anti-Gas Ab (generated in the lab of Dr. Lee S. Weinstein) (1:1000)
Rabbit anti-HA Ab (Cell Signaling #3724) (1:1000)

The following secondary antibodies were used:
Alexa Fluor 488 goat anti-guinea pig secondary Ab(Thermo Fisher Scientific, #A-11073)(1:500)
Alexa Fluor 488 goat anti-mouse secondary Ab (Thermo Fisher Scientific, #A-11017) (1:500)
Alexa Fluor 488 goat anti-rabbit secondary Ab (Thermo Fisher Scientific, #A-11034) (1:500)
Alexa Fluor 555 goat anti-guinea pig secondary Ab (Thermo Fisher Scientific, #A-21435) (1:500)
Alexa Fluor 594 goat anti-mouse secondary Ab (Thermo Fisher Scientific, #A-11032) (1:500)
Alexa Fluor 594 goat anti-rabbit secondary Ab (Thermo Fisher Scientific, #A-11037) (1:500)
Anti-mouse IgG, HRP-linked secondary Ab (Cell Signaling, #7076) (1: 5000)
Anti-rabbit IgG, HRP-linked secondary Ab (Cell Signaling, #7074) (1:5000)

Validation

Validated by the manufacturer (for details, please visit the manufacturers' web sites)

Eukaryotic cell lines

Policy information about cell lines and Sex and Gender in Research

Cell line source(s)

alpha-TC6 cells (obtained from Dr. Rohit Kulkarni, as stated in the text)

Authentication

authenticated by ATCC

Mycoplasma contamination

This cell line tested negative for mycoplasma contamination.

Commonly misidentified lines
(See ICLAC register)

None

Animals and other research organisms

Policy information about studies involving animals; ARRIVE guidelines recommended for reporting animal research, and Sex and Gender in Research

Laboratory animals

Male mice (C57BL6 background) older than 8 weeks; see the Methods section for detailed housing conditions

Wild animals

No wild animals were used in the study.

Reporting on sex

Male mice were used since male mice are not subject to hormonal cycles.

Field-collected samples

No field-collected samples were used in the study.

Ethics oversight

All animal studies were approved by the NIDDK institutional Animal Care and Use Committee.

Note that full information on the approval of the study protocol must also be provided in the manuscript.

Plants

Seed stocks

N/A

Novel plant genotypes

Describe the methods by which all novel plant genotypes were produced. This includes those generated by transgenic approaches, gene editing, chemical/radiation-based mutagenesis and hybridization. For transgenic lines, describe the transformation method, the number of independent lines analyzed and the generation upon which experiments were performed. For gene-edited lines, describe the editor used, the endogenous sequence targeted for editing, the targeting guide RNA sequence (if applicable) and how the editor was applied.

Authentication

Describe any authentication procedures for each seed stock used or novel genotype generated. Describe any experiments used to assess the effect of a mutation and, where applicable, how potential secondary effects (e.g. second site T-DNA insertions, mosiacism, off-target gene editing) were examined.
